# Reversal Learning and Associative Memory Impairments in a BACHD Rat Model for Huntington Disease

**DOI:** 10.1371/journal.pone.0071633

**Published:** 2013-11-01

**Authors:** Yah-se K. Abada, Huu Phuc Nguyen, Bart Ellenbroek, Rudy Schreiber

**Affiliations:** 1 Neuropharmacology, EVOTEC AG, Hamburg, Germany; 2 Brain Research Institute Dept. of Neuropharmacology, University of Bremen – FB 2, Bremen, Germany; 3 Institute of Medical Genetics and Applied Genomics, University of Tübingen, Tübingen, Germany; 4 School of Psychology, Victoria University of Wellington, Wellington, New Zealand; 5 Behavioral Physiology & Pharmacology, University of Groningen, Groningen, The Netherlands; National Center of Neurology and Psychiatry, Japan

## Abstract

Chorea and psychiatric symptoms are hallmarks of Huntington disease (HD), a neurodegenerative disorder, genetically characterized by the presence of expanded CAG repeats (>35) in the HUNTINGTIN (HTT) gene. HD patients present psychiatric symptoms prior to the onset of motor symptoms and we recently found a similar emergence of non motor and motor deficits in BACHD rats carrying the human full length mutated HTT (97 CAG-CAA repeats). We evaluated cognitive performance in reversal learning and associative memory tests in different age cohorts of BACHD rats. Male wild type (WT) and transgenic (TG) rats between 2 and 12 months of age were tested. Learning and strategy shifting were assessed in a cross-maze test. Associative memory was evaluated in different fear conditioning paradigms (context, delay and trace). The possible confound of a fear conditioning phenotype by altered sensitivity to a ‘painful’ stimulus was assessed in a flinch-jump test. In the cross maze, 6 months old TG rats showed a mild impairment in reversal learning. In the fear conditioning tasks, 4, 6 and 12 months old TG rats showed a marked reduction in contextual fear conditioning. In addition, TG rats showed impaired delay conditioning (9 months) and trace fear conditioning (3 months). This phenotype was unlikely to be affected by a change in ‘pain’ sensitivity as WT and TG rats showed no difference in their threshold response in the flinch-jump test. Our results suggest that BACHD rats have a profound associative memory deficit and, possibly, a deficit in reversal learning as assessed in a cross maze task. The time course for the emergence of these symptoms (i.e., before the occurrence of motor symptoms) in this rat model for HD appears similar to the time course in patients. These data suggest that BACHD rats may be a useful model for preclinical drug discovery.

## Introduction

Huntington disease (HD) is one of the neurodegenerative disorders where the origin has been unequivocally identified, that is, an elongation of polyglutamine (>35 CAG repeats) in the *HUNTINGTIN* (*HTT*) gene on chromosome 4 [1]. Patients carrying the mutation present a combination of motor symptoms such as chorea, psychiatric symptoms, and cognitive changes [2]–[3]. The disease is associated with degeneration of neurons in the striatum (especially the Medium Spiny Neurons, MSN) and cortex [4]–[6]. Treatments to delay HD onset or inhibit the mechanisms by which neural loss occurs are still lacking [7], and therefore there is a continuing need for improved animal models to support drug discovery efforts.

During the last decades, many animal models for HD have been generated, from insects (*Drosophilae melanogaster*), to nonhuman primates (*Macaca mulatta*), including several rodents models [8]–11. The availability of such a wide range of models increases the potential opportunities to understand the disease progression and to find a cure. Besides selection of a reliable and valid animal model, the timing of drug treatment is of critical importance for HD drug discovery studies. One plausible explanation for the recent failure of monoclonal antibodies against the beta-Amyloid protein to reverse symptoms in patients with advanced Alzheimer's disease in Phase III studies, has been that therapeutic intervention is needed at a time point when the disease has not yet caused too much neurodegeneration for treatments to be effective [12]. Accordingly, it is increasingly recognized that identification and validation of prodromal symptoms and biomarkers is critical. For HD, cognitive impairments may consist of prodromal symptoms that could be used as clinical endpoints in drug discovery. HD patients present several impairments in executive and visuospatial mnemonic functions [13]–[15]. Cognitive impairment appears to occur *before* the emergence of motor symptoms. For example, patients exhibited impairments in the California verbal learning test (CVLT) and the Wechsler memory scale (WMS)] in the absence of motor disturbances [16]. An important aim for future animal model development is to identify, characterize and validate cognitive symptoms that occur *before* the onset of motor symptoms. It is not yet clear to what extent the occurrence of cognitive and motor symptoms are adequately reflected in the current rodent HD models. In fact, several studies reported cognitive impairments *after* the appearance of motors deficits. For example, in a tgHD rat model of HD that carry the human mutation with 51 CAG repeats [17], Fielding and colleagues [18] have not found significant impairment in object recognition, set shifting, and operant tests, although motor deficits were present at 13 months of age. Deficits were shown at 12 months of age in radial maze and at later ages (15 to 20 months old rats) in choice reaction time tasks, spatial, and location recognition memory tests, whereas in the R6/2 mouse model for HD, selective deficits in spatial, visual and reversal discrimination were observed before or during subtle motor deficits (3.5–5.5 weeks and 7–8 weeks respectively) [19]–[24].

Herein, we used BACHD rat, a novel model for HD that has been recently established [25]. Like the mouse BACHD model [26], the rat model carries the full length human mutant *HUNTINGTIN* (fl-*mHTT*) with 97 CAG-CAA mix repeats under control of the human HD promoter gene. An advantage of the rat model is that behavioral processes related to learning and memory and pharmacological validation have been well described for this species. We have previously found progressive motor deficits during rotarod testing, starting as early as 2 months of age; a decrease in spontaneous locomotor activity, as well as, gait deficits in a catwalk test. However, we were unable to show a significant cognitive impairment in an object recognition task or robust sensorimotor deficits in a prepulse inhibition test [27]. These results were somewhat unexpected in light of the cognitive deficits reported in patients. Therefore, we decided to perform a more profound evaluation of the cognitive phenotype of BACHD rats. The cognitive performance of different age cohorts of BACHD rats was assessed in reversal learning and associative memory tests. The cross-maze and the fear conditioning paradigms tests (contextual, delay and trace conditioning) were selected, because they have proven to be efficient for cognitive assessment in rodents. An important consideration for selection of the rat fear conditioning paradigms was that the neural circuitry for fear conditioning has been well described in rodents and humans and that these neural circuits are well conserved [28]–[29]. This offers a potentially powerful translational approach as fear conditioning studies in tandem with functional brain imaging studies in both species could be used for future drug discovery studies.

## Materials and Methods

### Ethics statement

The study was carried out in strict accordance with the German animal welfare act and the EU legislation (EU directive 2010/63/EU). The protocol was approved by the local ethics committee *Behörde für Gesundheit und Verbraucherschutz* (BGV, Hamburg).

### Husbandry and genotyping

Wild type (WT) and transgenic (TG) BACHD rats, carrying the mutant human HTT gene, under the control of the human huntingtin promoter and its regulatory elements were used. The transgene contains 97 CAG-CAA mix repeats, which produces a particular stability of the repeat length, and additional 20 kb upstream and 50 kb downstream sequences that reduce its position effect [25]. Two transgenic males were supplied from the original BACHD colony of the Universitäts Klinikum Tübingen (UKT, Germany) and an in-house breeding colony was preserved and maintained at EVOTEC AG (Hamburg, Germany) by cross-breeding these males with wild type female rats. BACHD animals were maintained on a Sprague-Dawley background. All the animals at weaning were group-housed 2 to 4 per cage with wood shavings and a filter top. The environment was enriched with a play tunnel and shredded paper. BACHD rats were maintained in climate controlled housing, with a 12-h reversed dark/light cycle (light from 19∶00 to 07∶00). Rats had free access to food and water except during experiments.

Ear punches were taken at weaning to determine their genotype. Genotyping was performed before and after all the studies using a validated protocol. Briefly, Genomic DNA was prepared from ear biopsy tissue using proteinase K digestion, followed by phenol/chloroform extraction (Qiagen DNeasy Tissue kit). Primers flanking the polyQ repeat in exon 1 were designed to recognize whether or not the rat carried at least one copy of the mutation, and were used to PCR amplify the polyQ regions [Q3: 5′ – AGG TCG GTG CAG AGG CTC CTC - 3′ and Q5: 5′ – ATG GCG ACC CTG GAA AAG CTG - 3′]. Gene status was confirmed in parallel by using designed primers from UKT [exon 1: FW 5′-ATG GCG ACC CTG GAA AAG CTG- 3′ and RV: 5′ -AGG TCG GTG CAG AGG CTC CTC- 3′; exon 67: FW 5′-TGT GAT TAA TTT GGT TGT CAA GTT TT- 3′ and RV: 5′ –AGC TGG AAA CAT CAC CTA CAT AGA CT- 3′]. The PCR product was run on an automated apparatus PTC-200 (Peltier Thermal Gradient Cycler) and the Agilent 2100 Bioanalyser (Agilent technologies) was used to determine the fragments size.

Our concern in this longitudinal study was to reduce as much as possible potential confounds that hamper the interpretation or extrapolation of the results. Therefore only male rats were used in the cognitive tests as the female estrus cycle may influence experimental outcomes [30]–[31].

### Strategy and shifting (Cross-Maze)

The strategy shifting test is a standard dual-solution task which was used to assess the respective contributions of response (or egocentric) and place (or allocentric) learning strategies on memory [32]. It determines the relative involvement of these 2 strategies during the course of learning. We essentially used the same method as has been described for testing the BACHD mice [33].

Spatial alternation was assessed using a modified version of the standard cross-maze; the home made maze consists of 4 identical arms (50 cm×12 cm×20 cm) at 90 degrees to each other. The maze was made with clear Plexiglas, elevated 45 cm above the floor, and a T-maze was created by closing one arm (north, N) with a guillotine door. The T-maze configuration was as follow: 2 arms [east (E) and west (W)] are at 180 degrees to each other, and the last arm (south, S) was perpendicular to these arms. Two holes were present: one at the end of the E and W arms each, and spatial cues were placed on a black curtain which surrounded the maze. A home cage was put at one end of the arms (E or W) to motivate the animals to explore the maze and find the exit into this home cage (where they were additionally rewarded with food pellets). One week prior to the test, rats received small sucrose food pellets in addition to their normal diet. One day prior to the start of the experiments, all rats received a 5-min habituation session in the apparatus. During that period, food was not available.

The next day, the acquisition sessions started and a rat was placed in the S arm. The home cage containing sucrose food pellets was placed under the hole in the W arm. The rat had to guide itself in the maze and reach the home cage. During acquisition, rats received one trial per day for 7 days. During the first 2 days, the goal arm (i.e. arm giving access to the home cage) was baited with small sucrose food pellets. The same training procedure was run during reversal and extended reversal training sessions except that the rats had to reach this time the home cage placed underneath the E arm (opposite of the previously learned arm). When a rat made a wrong choice (entrance into the arm without home cage), it was allowed to trace back to the goal arm. If the rat failed to reach the home cage within 2 min, the rat was gently guided manually to the goal arm and the trial ended 20 s later. The maze was cleaned after each animal crossing with a 10% ethanol solution to avoid any bias related to odor.

Three probe trials were performed at days 8, 16 and 22, at the end of the acquisition, reversal and extended reversal training sessions, respectively. During the probe trials the S arm was closed and the N arm was used as the new start arm; the strategy (place or response) that the animal used to reach the goal arm was assessed. If the animal during the acquisition training had learned to use a place strategy, it would select the W arm. However, if the animal had used a response strategy (i.e. learned to turn left), it would select the E arm.

### Fear conditioning

Classical fear conditioning (FC) is a form of associative learning in which subjects express fear responses to a neutral conditioned stimulus (CS) after it has been paired with an aversive, unconditioned stimulus (US). The tests were run in an apparatus (Med Associates Inc., Italy) consisting of a ventilated sound-attenuated box and a rectangular testing chamber (30×26×25 cm) with stainless steel rod floor. Measurements were accomplished through a front digital video recording camera, connected to a computer with video freeze software. All rats received 5 min acclimatization one day prior to training and testing days. The chambers were wiped with a 70% ethanol solution and were dried prior to each rat testing. Three different tasks were used: contextual, delay and trace fear conditioning.

#### Contextual fear conditioning

The training session consisted of a 5 min acclimatization followed by 6 pairings (1 min inter trial time) of a 0.6-mA, 1-s foot shock. Animals were returned to their home cage 3 min after receiving the last foot-shock. On the next day, conditioned freezing was assessed by placing rats in the conditioning chambers for 5 min, in the absence of foot shock. For the evaluation of long term memory (LTM), animals were re-exposed one and 2 months later during a 5-min sessions to the conditioning chambers.

#### Delay conditioning

The testing protocol is similar to the contextual fear paradigm, except that on the training day, after 3 min acclimatization, rats received 6 pairings (120 s inter trial time) of a 30-s tone (85 dB) with a 0.6-mA, 2-s foot shock. The foot shock terminated at the same time as the tone and rats were removed from the testing chambers 60 s after the last pairing. On the testing day, rats were tested for contextual freezing in the conditioning chambers for 3 min, in the absence of tone or foot shock. One hour later, an altered context was generated with white polyvinyl chloride materials that covered the shock-grid bars and the inside of the conditioning boxes. Freezing was assessed in the altered context without tone for 3min, followed by a 3-min tone presentation in the absence of foot shock.

#### Trace fear conditioning

The test was adapted from an existing protocol [34]. In this associative learning paradigm, rats received during the training day eight trials of a 85 dB, 10 s tone (CS), followed by 20 s trace period, after which a 1 s–0.6 mA foot shock (US) was delivered. Each CS-US pairing was separated by a random inter-trial interval (ITI) that varied between 60 and 120 s. The random ITI time was used to prevent time between foot-shocks to be used as a cue for the US. Rats were removed from the chamber 60 s after the last CS-US presentation.

Retention tests for contextual, auditory and trace fear memory were carried out 24 h after conditioning. Rats were first tested for tone and trace period in an altered context made with a white polyvinyl chloride insert to cover the shock-grid bars and the inside of the conditioning boxes. Each rat was given 2 min habituation, followed by four presentation of the CS with varied ITI, in the absence of US. Freezing behavior during the four CS presentations and trace periods were averaged for each animal. Following CS and trace retention testing periods, contextual retention test was measured by placing the animals back into the original context for 2 min during which freezing was scored, without exposure to the CS or US.

For all the paradigms, freezing behavior was defined as the lack of any movement, except respiration. The percent of time spent freezing was assessed using the linear methods of observation measures (video freeze software).

### The ‘flinch-jump’ test

The method has been described by Lehner and colleagues [35]. Rats were placed individually into the fear conditioning boxes (Med Assoc. Italy). Shocks were delivered to the grid floor of the test box through a shock generator. After a 3-min period of habituation to the test box, shock titrations continued to increase in a stepwise manner (0.05 mA, 0.05–0.6 mA range). In this way, the ‘flinch’ and ‘jump’ thresholds in mA is defined for each rat. The interval between shocks was 2 min, and each animal was tested only once at each intensity. Behavior for each rat was recorded through a front digital video recording camera and analysis was done blind to the genotype. The ‘flinch’ threshold was defined as the lowest shock intensity that elicited a detectable response. The ‘jump’ threshold was defined as the lowest shock intensity that elicited simultaneous removal of at least three paws (including both hind paws) from the grid.

### Statistical Analysis

All data were analyzed using *GraphPad* and *InVivoStat* software. Differences between groups were assessed with Student's t-test or mix ANOVA with repeated measures, with the factor GENOTYPE as between subject and TIME or TEST as within subject variable. When significance was found, a Bonferroni – post hoc analysis was performed when appropriate. For the cross maze, the learning index is defined as the ratio of the mean number of correct choices over trials per animal. Therefore, we have generated a binary data set with 2 possible outcomes (correct choice vs. incorrect choice). The hypothetical value that results is “½” because each animal has 50% chance at every trial. The one sample t-test was used to evaluate the learning index in each population with a hypothetical value set at “½”. Chi-square (χ2) analyses were computed on animal's choice during Acquisition (A), Reversal (R), and Extend Reversal (ER) learning in the cross maze test, in order to determine discrepancies between groups and to determine potential changes in strategies between both probe trials. The Chi square test assesses whether an observed frequency distribution (i.e. the number of correct choices) differs from a theoretical distribution, and if this distribution is independent (i.e. the choice is genotype dependent). Finally, a Mann Whitney U-test was used to analyze ‘flinch-jump’ data. The significance level was set for all analysis at 0.05.

## Results

We inspected each BACHD rat cohort animals prior to the experiments and all animals looked healthy. No global differences in phenotype were observed between wild type (WT) and transgenic (TG) rats. Only male rats were used during the study. No difference in weight was found between WT and TG (data not shown).

### Acquisition, Reversal learning and Strategy shifting in a Cross-maze

**Figure 1:**
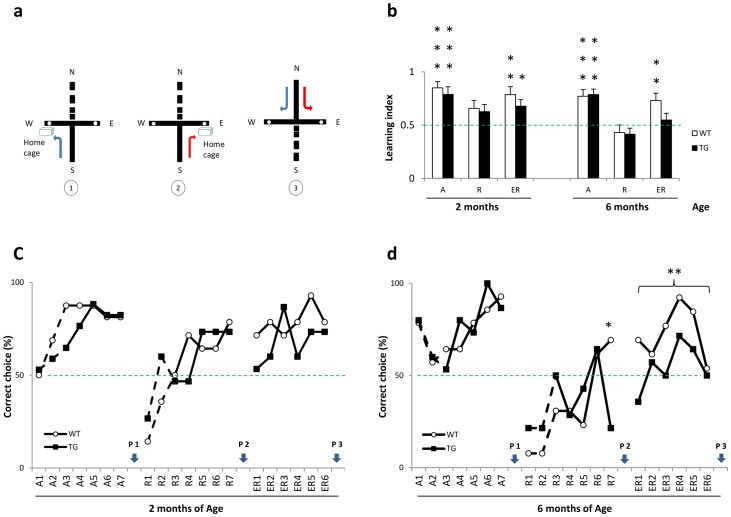
Cross-maze task. 2(WT n = 17, TG n = 17) and 6 months (WT n = 15, TG n = 15) BACHD rats were used. [**a**] Schematic representation of the cross-maze task. The north (N) arm is closed. The rat starts training in the south arm (S) and reaches the home cage through the hole located in the west arm (w, (1) acquisition) or east arm (E, (2) reversal). During (3) probe trial days 8 (P1), 16 (P2) and 23 (P3), the (S) arm is closed and the rat starts in the (N) arm. Rats reaching the home cage arm are Place learners, while those reaching the other arm are Response learners. [**b**] Learning index. Mean number of correct choices over acquisition (A), reversal (R) and extended reversal (ER) trials in BACHD rats. Both WT and TG rats of each age showed difficulties during (R); however, with (ER) training, 2 months old rats have improved learning whereas 6 months old TG rats have a learning index barely above chance level. Asterisks indicate significant difference from the hypothetical value (One sample test, *p<0.05, **p<0.01 and ***p<0.001). [**c** and **d**] Training trials. The percentage of correct choices made during acquisition (A), reversal (R) and extended reversal (ER) training are depicted. For the first 2 days, results where the goal arm was baited with sucrose food pellets are presented by dashed lines. There was no difference in acquisition training for both age cohorts. 6 months old TG rats (d) differed significantly from WT rats during reversal trial 7 and overall extended reversal trials (ER1 to ER6). Asterisks indicate significant difference (Chi square test, *p<0.05 and **p<0.01).

The cross maze test was performed in independent cohorts of 2 months (n = 17 per genotype) and 6 months (n = 15 per genotype) old BACHD rats in order to avoid any bias related to recall or long term memory of the task. Data from acquisition and reversal training sessions at the first 2 days were not analyzed because the goal arm was baited with sucrose food pellets to guide BACHD rats to the home cage. A schematic representation of the cross-maze task is presented in Fig. 1a. One WT rat of 2 months was removed from the data analysis because it did not show any interest in the task and did not make a choice (turn left or right) during the experimental period.

### Learning indices during acquisition, reversal and extended reversal learning

The learning index is calculated as the ratio of the mean number of correct choices over trials per animal (Fig. 1b). Both cohorts of 2 and 6 months old BACHD rats displayed improved learning during *acquisition* (A) (2 months: WT, t = 5.91 and P<0.0001; TG, t = 4.02 and P<0.001; 6 months: WT, t = 4.35 and P<0,001; TG, t = 5.776 and P<0.0001). During *reversal* (R), 2 months old WT and TG have a learning index above the chance level of 0.5, although both groups did only reach a statistical trend (p value between 0.05 and 0.1; 2 months: WT, t = 2.126 and P = 0.0532; TG, t = 1.884, and P  = 0.0805). The 6 months old cohort (WT and TG) showed a (R) learning index below chance level (<0.5) and did not reach statistical significance. In the *extended reversal* training (ER), both WT and TG rats of 2 months presented a statistically significant learning index (WT, t = 3.809 and P<0.01; TG, t = 2.874 and P<0.05). Six Months old WT rats had a significant learning index during (ER) (t = 3.323, and P<0.01) whereas TG did not reach statistical significance (t = 0.743, and P>0.1); 6 months old TG learning index was around 0.5. A comparison between the (A) and (ER) learning index in WT and in TG rats showed only a difference for 6 months old TG rats (A vs. ER, t = 2.846, P<0.01). A closer look at the 6 months rats (ER) bar graph (Fig. 1b) suggests a difference between WT and TG rats, and statistical analysis found a trend (t = 1.737, P  = 0.0946).

### Correct choices during acquisition, reversal and extended reversal learning

The progress of 2 and 6 months old BACHD rats in learning the task during training sessions for (A), (R) and (ER) is presented in Fig. 1 (c–d). We analyzed the number of correct choices that both groups made. All rats had one trial per day. There was no statistical difference during (A) in 2 and 6 months old BACHD rats. For (R) and (ER), only animals with learning index higher than 0.5 during acquisition were analyzed. That is, animals that actually learned the task. Four (WT, n = 2 and TG, n = 2) 2 months old rats and two (WT, n = 1 and TG, n = 1) 6 months old rats did not reach the criteria and therefore were not included in the analysis. Although 2 months old TG rats made correct choices during (R) and (ER) training, as did WT control rats, 6 months old TG rats made fewer correct choices. In fact, WT – but not TG – animals made more correct choices over the course of (ER) training. The difference between 6 months WT and TG rats was significant already on reversal trial 7 (χ2 = 6.23, P<0.05) and persisted during (ER) trainings (χ2 = 8.594, P<0.01).

### Strategy shifting during probe trials

**Figure 2:**
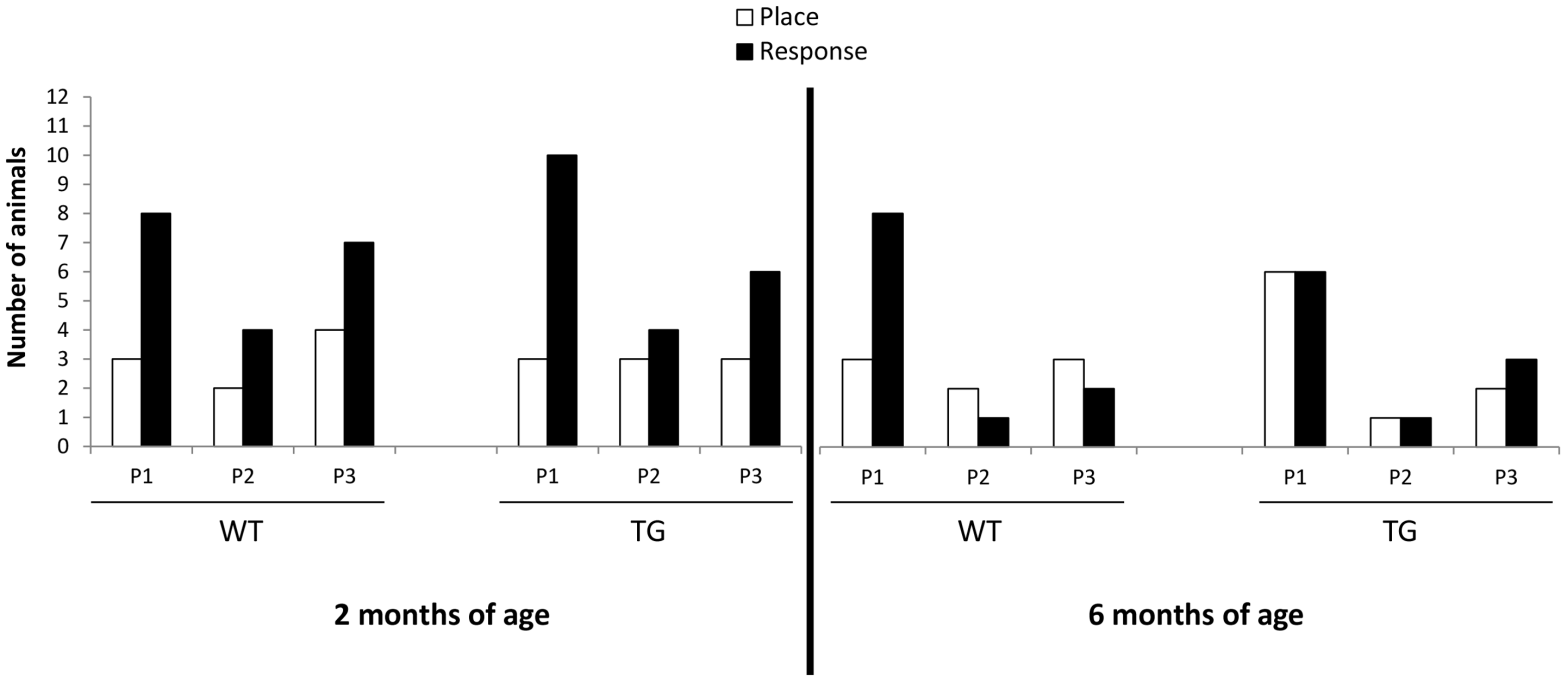
Strategy shifting. Number of rats that exhibited Place (P) or Response (R) learning strategy during each Probe trial P1, P2 and P3 (days 8, 16 and 23 respectively) are represented for WT and TG cohorts of 2 and 6 months of age. The size corresponds to animals that made (1) correct arm choices with a learning index greater than 0.5 during each training session, and (2) were successful for the two last trials prior to the probe trials in the cross maze.

On trial days 8, 16, and 23 a probe trial was done to assess which strategy rats used to solve the task (Fig. 2). The north arm (N) was now the new start arm. Rats entering the same arm as during training sessions were designated place learners (allocentric learning) and rats entering the opposite arm were designated response learners (egocentric learning). Data were only analyzed for animals that made (1) correct arm choices with a learning index greater than 0.5 during each training session, and (2) were successful for the two last trials prior to the probe trial. Two months old rats exhibited a preference for response learning on P1 (*response:* WT = 73% and TG  = 77%) and P2 (*response:* WT  = 67% and TG  = 57%). This preference for response learning was maintained on P3 (*response:* WT  = 64% and TG  = 67%). Six months old WT rats again exhibited a clear response learning during P1 (WT  = 73%) while only half of TG rats were response learners. However, during P2 and P3, WT rats have adopted a place learning strategy (WT, *place:* P2 = 66% and P3 = 57%), whereas TG rats showed a response learning strategy (TG, *response:* P2 = 50% and P3 = 60%). Although WT rats results between both probe sessions (P1→P3) would suggest a shifting towards a place learning (Δ = 30%), this was not statistically significant (χ2 = 1.606, P>0.05).

### Contextual fear conditioning

**Figure 3:**
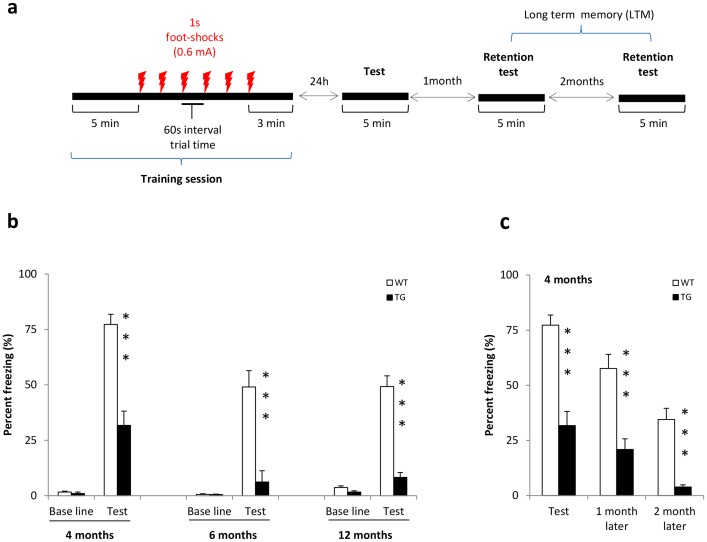
Contextual fear conditioning. [**a**] Schematic illustration of the contextual fear conditioning protocol. [**b–c**] Results are expressed as Mean ± SEM of percentage freezing. 4 months (WT n = 13, TG n = 13), 6 months (WT n = 7, TG n = 9) and 12 months (WT n = 16, TG, n = 6) BACHD rats were used. No difference in baseline responding to training was observed. TG rats showed less fear memory to the context as they freeze less in comparison with WT rats at 4, 6, and 12 months of age [**b**]. Long term memory was assessed 1 month and 2 months after retention testing were conducted in the 4 months old rats cohort (i.e. they were tested at 5 and 6 months of age respectively) [**c**]. TG compared to WT still had lower freezing to the context. A progressive freezing ‘extinction’ was observed. Asterisks indicate significant differences between WT and TG rats (***p<0.001).

Rats of 4 months (WT, n = 13 and TG, n = 13), 6 months (WT, n = 7 and TG, n = 9) and 12 months (WT, n = 16 and TG, n = 6) of age underwent a one day training session in conditioning chambers. Baseline activity was recorded 5 min before foot shocks were given and contextual memory was measured 24h later (Fig. 3a).

As shown in Fig. 3b, there was no significant difference between WT and TG baselines at all testing ages. However, TG rats expressed a significant lower freezing behaviour when re-exposed to the conditioning context (4 months: t = 5.757, P<0.0001; 6 months: t = 4.987, P<0.001 and 12 months: t = 5.147, P<0.0001). Visual inspection of fig. 3b indicates a decrease in percentage of freezing between 4 months, 6 and 12 months old rats. In fact, a 2-way ANOVA analysis on Context results showed significant GENOTYPE (F (1, 79)  = 76.53, P<0.001) and AGE (F (2, 79)  = 13.42, P<0.001) effects. No interaction between GENOTYPE x AGE was found.

We next evaluated long term memory for contextual freezing in 4 months old rats by exposing them again to the conditioning chambers 1 and 2 months after the contextual test (retention tests, Fig. 3c). A progressive ‘*extinction*’, characterized by a decrease in percentage freezing was observed in WT and TG rats (2-way ANOVA, GENOTYPE: F (1, 72)  = 82.92, P<0.001 and AGE: F (2, 72)  = 24.48, p<0.001). This trend is sustained as no interaction (GENOTYPE x AGE) was found.

### Delay and Trace conditioning

**Figure 4:**
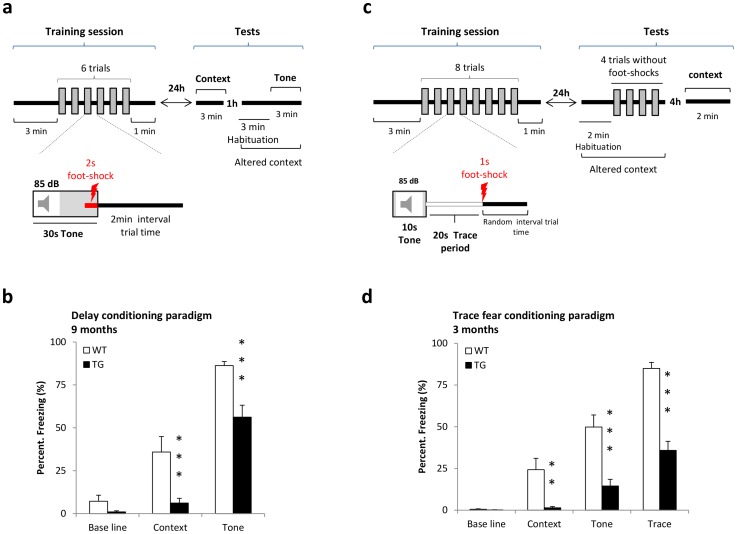
Delay and Trace fear conditioning. [**a**] Schematic illustration of the delay fear conditioning paradigm. [**b**] Results in 9 months old BACHD rats are expressed as Mean ± SEM (WT n = 13, TG n = 15). TG rats presented a significant lower freezing response to the context and to the tone. [**c**] Schematic illustration of trace fear conditioning paradigm. [**d**] Results in 3 months old BACHD rats are expressed as Mean ± SEM (WT n = 13, TG n = 15). TG rats, compared to WT rats, showed a significantly lower freezing response to the context, to the tone and trace period during retention tests. Asterisks indicate significant differences between WT and TG rats (**p<0.01 and ***p<0.001).

The delay conditioning experiment evaluated the acquisition of a tone (85dB) fear conditioning when presented for 30 s before a 2 s foot-shock co-termination (Fig. 4a). Thirteen WT and fifteen TG rats of 9 months of age were given 6 trials training sessions. Baseline activity was recorded 3 min prior to the first trial and expressed as percentage freezing. WT and TG rats did not show differences in baseline freezing behavior (Fig. 4b). A 3-min retention test was performed after 24 h in the conditioning context and, for the tone, in an altered context. Both WT and TG rats expressed a trend for increased freezing to the context and the tone. TG rats showed a lower percentage freezing to the context and to the tone than WT rats. A 2-way ANOVA revealed significant effects for the main factors GENOTYPE and TEST (GENOTYPE, F (1,52)  = 18.84, P<0.001; TEST, F (2,52)  = 132.04, P<0.0001) as well as a significant interaction between both factors (F (2,52)  = 5.04, P<0.01).

In the trace fear conditioning paradigm, the memory for context, tone and trace training is evaluated in 3 months old rats (Fig. 4c). The highest freezing responses were found during the trace period (Fig. 4d). No differences in baseline activity were found, but for all the stimuli (context, tone and trace), TG rats displayed a significantly lower freezing response than WT rats. A 2-way ANOVA analysis showed significant effects for the main factors GENOTYPE and TEST (GENOTYPE, F (1,78)  = 39.34, P<0.0001; TEST, F (3,78)  = 96.42, P< 0.0001), as well as a significant interaction between both factors (F (3,78)  = 15.08, P<0.0001).

### Flinch-Jump test

**Figure 5:**
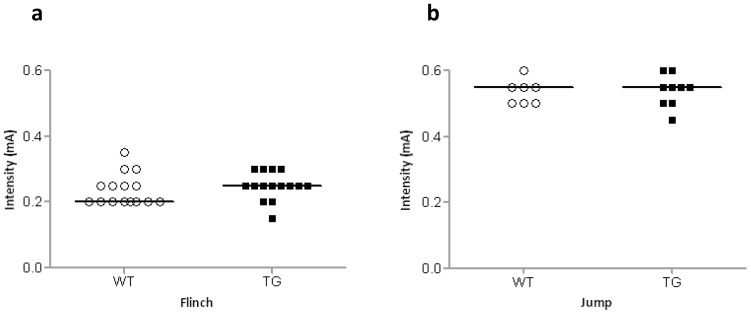
Flinch-jump test. Sensitivity of 6-shocks for [**a**] flinch and [**b**] jump (WT n = 15, TG n = 15). Individual intensity response is plotted and bars indicate median values for each genotype. There was no difference between WT and TG rats in current intensities that elicited a flinch or a jump response.

Thirty BACHD rats (n = 15 per genotype) of 6 months of age underwent the flinch-jump test. A flinch response was observed in all rats (Fig. 5a. WT, Mean  = 0.23±0.048 SEM; TG, Mean  = 0.25±0.042 SEM); whereas only 7 WT and 9 TG rats presented a jump response (Fig. 5b. Mean  = 0.535±0.037 SEM and Mean  = 0.538±0.048 SEM, respectively). In fact, statistical analysis did not reveal significant differences between WT and TG rats (Mann Whitney U-test: flinch, U = 85 and P = 0.23; Jump, U = 29 and P = 0.82).

## Discussion

We investigated the cognitive phenotype of BACHD rats at ages 2 through 12 months. Learning deficits were found at 6 months of age in a cross-maze test. Pronounced associative memory deficits were found in context, delay (context and tone) and trace (context, tone and trace) fear conditioning. This fear conditioning phenotype is unlikely to be confounded by altered pain sensitivity, as WT and TG rats showed no differences in foot-shock intensity threshold as determined in a flinch-jump test. This is the first study to report robust and specific memory deficits in BACHD rats.

### Reversal learning deficit in cross maze test

We have investigated BACHD rats in a spatial memory paradigm with the cross maze test. A T-maze standard dual solution task [32] has proven to be useful in distinguishing between spatial and non spatial learning in animals. Rats were trained over several trials from the same start arm to consistently enter the arm where a baited home-cage was located. We used a home-cage baited with sucrose pellets as an alternative to traditional motivational procedures that use water or food deprivation. Since BACHD transgenic rats show reduced food intake [25], we felt that procedures that avoid food deprivation may be less liable to potential confounds and misinterpretation of behavioral data.

During Acquisition training (A) both TG and WT rats learned the task and showed a significant learning index (∼0.8). This indicates that a ‘return to home-cage’ is an effective incentive and that a training protocol of only one trial per day is sufficient. These observations are in line with results from a study in adult B6D2F_2_ mice (cross of C57BL/6J and DBA/2J strains) in a Lashley III maze. It was demonstrated that one training trial per day with a home-cage reward procedure led to a significant learning index (∼0.7) after just 4 days [36].

During Reversal training (R), the home cage was located at the end of another arm, different from the initially trained arm. The same start arm was used. *All* rats of 2 and 6 months of age initially had difficulties finding the new location. This was confirmed by a comparison of the percentage correct choice of the last two acquisition trials (6 and 7) with the first two reversal trials (1 and 2). A difficulty in finding the new location is perhaps not surprising, as reversal learning is more challenging per se because rats have to disengage from a previous learned task in order to acquire a new task. We decided to extend the reversal training for 6 days (Extended Reversal training; ER) and all rats eventually learned the new task, although the 6 months old TG rats performed significantly worse than WT rats. We previously reported similar reversal learning deficits in adult transgenic BACHD mice of 10 months of age in a cross-maze task [33]. In 6 months old Hdh^(CAG)150^ knock-in mice, cognitive impairments were shown in compound reversal of an extra-dimensional shift task (EDS) [37]. Reversal learning difficulties were also reported in a spatial operant reversal test paradigm of 9 months old tgHD rats and in 27-week old YAC128 mice in a water T-maze task [38]–[40]. These data are in accordance with the present findings, suggesting a progressive cognitive decline between 2 and 6 months of age.

To discover which learning strategy rats have adopted, a test trial was performed after training was finished. The new start arm was now located opposite of the arm used during training. Accordingly, rats which used spatial cues to find the correct arm would enter the same baited arm as during trainings (place learners); whereas rats that used ‘body turn response’ learning (stimulus response, S-R) should enter the non baited arm (response learners). The first probe trial (P1) demonstrated that BACHD rats of 2 and 6 months of age were predominantly *response* learners. Using a similar cross maze task, this preference for the *response* strategy on (P1) was also observed in WT and TG BACHD *mice* [33]. Consistent with our findings, homozygote tgHD rats of 6 and 12 months of age were also mostly response learners in a Morris water maze task [41]. Interestingly, our results are in contrast with findings suggesting that during (A), *place* learning is typically adopted by rats in a cross-maze [32], [42]–[43]. The reason why 2 months old rats maintain their preference for response learning during P3 is unclear, but may involve a developmental time scale of spatial representation. In fact, spatial memory in the cross-maze involves association of the object (landmarks) to their spatial location (home cage); we assume that the network underlying the memory of spatial location in 2 months old WT rats is slower to develop [44]. The preference (shift) for *place* strategy was only observed in 6 months old WT during the 2^nd^ and 3^rd^ probe trial (P2 and P3), whereas TG rats maintained their *response* strategy. The same strategy was seen in adult BACHD mice during reversal probe trial [33]. The reason why BACHD rats maintain response learning may probably involve altered functioning of fronto-hippocampal (place learning) vs. fronto-striatal (response learning) circuitry [45]–[47]. Indeed, TG rats show already at 3 months of age abundant *htt* aggregates in the CA3 region of the hippocampus, whereas only few aggregates were present in the caudate-putamen [25]. Therefore, the striatal-based ‘body turn response’ might prevail during learning and subsequent probe testing in TG rats.

The rodent data are consistent with human data. Similar impairments in reversal learning and strategy, when attention has to be shifted from one perceptual dimension to another, have been demonstrated in early and advanced-stage HD patients in an extradimensional shift (EDS) test and in a Wisconsin Card Sorting Test (WCST). These patients made perseverative errors suggesting memory inflexibility [13]–[15], [48]. Finally, cognitive set shifting ability in EDS and WCST involves cortical and basal ganglia circuitry system, especially the prefrontal cortex and the caudate nucleus [49].

### Associative learning deficits in fear conditioning test

We performed an extensive characterization of BACHD rats in various fear conditioning paradigms and found very robust deficits across all age cohorts and under all experimental conditions. A technical challenge that was successfully mastered was the selection of an appropriate current intensity. One that was not too high – high intensities would lead to a generalized freezing response – or too low – low intensities would lead to large variability in freezing and inconsistent fear conditioning [50]. A confound of the BACHD fear conditioning phenotype by motor deficits seems unlikely since rats did not show any difference in percent of time freezing during habituation. In order to address if altered sensitivity to foot shocks (US) may have confounded the fear conditioning phenotype, we employed a flinch-jump test and found no differences between WT and TG rats. We used a relatively low shock intensity (0.6 mA) which may explain that not all rats showed a ‘jump’ reflex. Our data are consistent with findings in Wistar rats where no correlation was found between pain sensitivity, conditioned and novelty-evoked fear responses in ‘flinch-jump’, ‘tail flick’ and ‘contextual’ fear tests [35]. Together, these data suggest that the deficit in conditioned fear responses in BACHD rats are not confounded by motor deficits or altered sensitivity to foot shocks.

What are the mechanisms underlying the fear conditioning deficits in BACHD rats? Learning in contextual fear conditioning is thought to involve association of stimuli present in the conditioned chamber (texture, shape, dimensions) with the (US) itself. More complex stimuli may put a higher demand on effective learning and memory [51], and thus may affect subjects with impaired activity in fear conditioning circuitry to a larger extent than unaffected subjects. The amygdala and hippocampus are involved in complex stimuli learning [52] and BACHD rats show *htt* aggregates in both brain areas [25]. Therefore, TG rat's lower freezing response to the context could be associated to a hippocampal-amygdala dysfunction. Such a conclusion is consistent with our findings from delay and trace fear conditioning testing. As expected from a stimulus with a higher salience, all rats showed higher levels of fear conditioning to the tone than to the context. The BACHD rats showed again a fear conditioning impairment. During trace fear conditioning, the Tone and shock (US) are separated by a time interval and the Trace period becomes predictive of the (US). Using these more complex stimuli, BACHD rats showed a clear deficit in fear conditioning. Impairments in fear conditioning have also been reported in mouse models for HD. For example, 5 weeks old R6/2 mice showed less contextual freezing than their wild-type control, although no difference was observed in tone conditioning [53] (Bolivar et al. 2003). In addition, a reduced fear expression during extinction retrieval and a reinstatement of a fear conditioning in R6/2 mice was not associated with a weakness in CS-US, but with neuronal *hypoactivation* in the prelimbic cortex, a subregion region of the prefrontal cortex [54]. Four months old CAG140 Knock-In (KI) mice have shown an increased freezing response during training, but, again displayed no deficit in recall tone fear conditioning [55]. We reported that adult transgenic BACHD mice present *higher* freezing rates to the context and tone during retention testing, and attributed this impairment to emotional deficits [33]. The difference in fear conditioning phenotypes between BACHD mice and rats is surprising. However, in view of the robustness of the rat phenotype and the fact that we are eventually interested in the translation of these findings to humans, it would be more sensible to perform fear conditioning studies in a non human primate model for HD [9], rather than undertaking an effort to further characterize the mouse fear conditioning phenotype.

Interestingly, reversal learning impairments in a cross-maze appeared at 6 months of age, whereas associative learning and memory deficits in fear conditioning tasks were already present at 3 months of age. Matching the different onset of these deficits with the emergence of *htt* aggregates in brain areas involved in the circuitry underlying cross maze behavior and fear conditioning will be helpful to translate the findings from rodents to humans. Especially for fear conditioning the functional neuroanatomy has been well described [28]. Rodent data support a role for the amygdala in the acquisition of conditioned fear, whereas the hippocampus and the medial prefrontal cortex (mPFC) are required for consolidation of long-term memory [56]–[59]. Human functional magnetic resonance imaging (fMRI) studies in delay and trace fear conditioning, have demonstrated a role of the hippocampus and other brain regions that support working memory processes in encoding temporal information and maintaining the associative representation CS-US during trace intervals [29]. Wide spread *htt* aggregates have been observed in brain areas involved in fear conditioning such as the neocortex, hippocampus, and the amygdala of BACHD rats [25]. However, the behavioral effects occurred at an earlier age than the *htt* aggregates (12 months). It is possible that more subtle molecular and cellular deficits in the cortex, hippocampus and amygdala contribute to the early deficits in fear conditioning. Further studies should address the developmental mechanisms underlying the disease progression in BACHD rats.

## Conclusion

Our study is the first to provide evidence of progressive cognitive deficits in a transgenic BACHD rat model for HD. TG animals showed difficulties in associative learning at 3 months of age in a fear conditioning test, and impairments in spatial memory at 6 months of age, mainly in reversal training where attention has to be shifted from one set of learning to another. BACHD rats recapitulate some of the cognitive impairments seen in HD patients. The precise time course for development of the cognitive symptoms requires further studies in additional age cohorts. As fear conditioning deficits appeared already in the youngest cohort tested, animals of 1 and 2 months of age need to be tested to determine if the onset of the fear conditioning is similar to the onset of, for example, rotarod deficits that occur at 2 months of age [27]. Emergence of cognitive deficits before motor deficits might more closely mimic the time course in HD patients [16]. In conclusion: the robust fear conditioning phenotype offers a firm foundation for future studies aimed to further characterize the time course for the associative memory deficit and its underlying neural circuitry. In addition, this functional readout can be validated for drug discovery approaches that target *htt* aggregates, using, for example, adenovirus-based viral transfection methods against htt [60].
